# Methodological Validation of the PIROP Ultrasound-Based System for Measuring Peri-Implant Soft Tissue Thickness in a Clinical Setting

**DOI:** 10.3390/jcm15041581

**Published:** 2026-02-17

**Authors:** Jakub Hadzik, Paweł Kubasiewicz-Ross, Krzysztof Kujawa, Tomasz Gedrange, Marzena Dominiak

**Affiliations:** 1 Department of Dental Surgery, Faculty of Dentistry, Wroclaw Medical University, Krakowska 26, 50-425 Wroclaw, Poland; 2 MCIW—Wrocław Medical Innovation Center, Krakowska 26, 50-425 Wroclaw, Poland; 3 Statistical Analysis Centre, Wroclaw Medical University, Statistical Analysis Centre, K. Marcinkowskiego 2-6, 50-368 Wroclaw, Poland; 4 Department of Orthodontics, Carl Gustav Carus University Hospital Dresden, Technische Universität Dresden, D-01307 Dresden, Germany

**Keywords:** peri-implant soft tissue thickness, ultrasonography, ultrasound validation, clinical measurement, implant dentistry, methodological study

## Abstract

**Background/Objectives:** Accurate and reproducible assessment of peri-implant soft tissue thickness is an important methodological aspect of contemporary implant dentistry, particularly in longitudinal studies evaluating soft tissue dimensions. While ultrasound-based techniques offer a non-invasive and quantitative approach, their validity in peri-implant settings remains insufficiently documented. The objective of this study was to validate the PIROP ultrasound-based system for measuring peri-implant soft tissue thickness by comparing it with a direct clinical reference method. **Methods:** Peri-implant soft tissue thickness was assessed at 40 planned implant sites at two predefined time points: prior to surgical incision and three months after closed healing. Measurements obtained using the PIROP ultrasound-based system were directly compared with measurements performed following surgical incision using a calibrated periodontal probe. **Results:** Overall, the relative differences between ultrasound-based and direct clinical measurements were small, indicating comparable performance under standardized clinical conditions. The PIROP ultrasound-based system demonstrated good agreement with the reference method, with high intraclass correlation coefficients (ICC = 0.86–0.88). **Conclusions:** Within the limitations of this methodological validation study, ultrasound-based assessment demonstrated good agreement with direct clinical measurements, supporting its use as a reliable, non-invasive, and quantitative measurement approach in clinical studies and longitudinal designs requiring repeated evaluation of peri-implant soft tissue thickness.

## 1. Introduction

According to the 2017 World Workshop on the Classification of Periodontal and Peri-Implant Diseases and Conditions, peri-implant health is defined by the absence of clinical signs of inflammation, while no specific range of probing depths compatible with peri-implant health has been established [1]. In this context, the literature describes a wide range of clinical protocols for peri-implant tissue management, as well as various prosthetic components and emergence profile designs aimed at supporting tissue stability and hygiene, reflecting the breadth of current knowledge in this field [2,3,4,5].

Since the Zero Bone Loss Concept has been introduced, the thickness of peri-implant soft tissues has been recognized as a key determinant of crestal bone stability and long-term implant survival. Evidence shows that mucosal thickness below 2 mm predisposes the site to additional crestal remodeling as the biological width reestablishes, potentially compromising implant longevity. Conversely, soft-tissue thickness of at least 2–3 mm is consistently associated with more favorable peri-implant bone levels and reduced marginal bone loss [6].

Accurate assessment of peri-implant soft tissue thickness is considered an important factor in contemporary implant dentistry, as soft tissue dimensions have been associated with peri-implant health, esthetic outcomes, and long-term stability of implant-supported restorations [7,8]. Variations in soft tissue thickness may influence mucosal stability, susceptibility to recession, and biological responses around dental implants, underscoring the need for reliable and reproducible measurement methods [9,10].

Several techniques have been proposed for the clinical assessment of peri-implant soft tissue thickness. These include direct visual measurement following surgical incision, which is commonly regarded as a reference approach due to direct exposure of the tissue dimensions. Other invasive methods involve transgingival probing techniques, such as measurement after mucosal perforation using periodontal probes or endodontic instruments (e.g., files) inserted through the soft tissue until bone contact is achieved [11,12]. While these methods enable direct or near-direct assessment of tissue thickness, they are inherently invasive and may be influenced by factors such as tissue compression, probe angulation, and operator-dependent variability.

In addition to invasive clinical techniques, radiographic assessment using cone beam computed tomography (CBCT) has been proposed for the evaluation of crestal soft tissue thickness prior to implant placement [13]. CBCT-based analysis enables cross-sectional measurement of soft tissue dimensions and simultaneous assessment of adjacent hard tissue parameters. However, this approach requires radiographic exposure.

Non-invasive qualitative approaches have also been described for the assessment of soft tissue characteristics. Color-coded probes, such as the Colorvue probe (Hu-Friedy, Chicago, IL, USA), have been used to facilitate evaluation of soft tissue thickness based on tissue translucency or probe visibility through the mucosa [14,15]. These methods allow chairside, non-invasive assessment; however, they provide only qualitative or categorical information and do not yield direct quantitative measurements expressed in millimeters. As such, their applicability for precise soft tissue thickness evaluation is limited.

The PIROP ultrasonic device was initially introduced as a high-frequency A-scan system for non-invasive measurement of periodontal soft tissue thickness. Early technical and conceptual work by Bednarz [16] described the principles and potential clinical applications of this technology, while subsequent clinical studies demonstrated its reproducibility for gingival thickness assessment under standardized conditions. In particular, Gánti et al. [17] reported good intra-examiner repeatability of PIROP-based measurements when compared with invasive transgingival probing techniques. Ultrasound-based techniques have been introduced as non-invasive tools for the assessment of peri-implant soft tissue thickness, enabling repeated measurements without tissue penetration. In the context of both clinical research and longitudinal study designs, the availability of a non-invasive measurement method capable of providing precise and reproducible quantitative assessments is of particular importance [17,18,19]. High-frequency ultrasonography allows real-time evaluation of soft tissue dimensions; however, despite these advantages, the clinical validity of ultrasound-based systems for peri-implant soft tissue thickness assessment requires careful methodological evaluation against established reference methods.

A major limitation of the existing literature is the limited number of validation studies directly comparing ultrasound-based measurements with reference clinical methods under standardized conditions. While many studies report descriptive measurements or treatment-related outcomes, fewer investigations address measurement agreement and reliability, leaving the methodological robustness of ultrasound-based systems insufficiently documented.

In the present study, direct clinical measurement performed following surgical incision using a calibrated periodontal probe was considered a clinical reference method rather than a true gold standard. Therefore, the aim of this study was to methodologically validate the PIROP ultrasound-based system by comparing its measurements with those obtained using the reference clinical method at predefined time points, without assessing procedure-related outcomes.

The aim of this study was to evaluate the agreement between peri-implant soft tissue thickness measurements obtained using the PIROP ultrasound-based system and a direct clinical reference method following surgical incision.

It was hypothesized that no relevant differences would be observed between ultrasound-based and direct reference measurements of peri-implant soft tissue thickness.

## 2. Materials and Methods

### 2.1. Study Design

This validation study was conducted as a methodological substudy nested within an investigator-initiated randomized clinical trial evaluating soft tissue augmentation procedures around dental implant sites. Although data were collected from participants enrolled in a randomized clinical trial, the present manuscript does not report the randomized clinical trial itself and does not assess treatment effects or clinical outcomes.

The objective of this substudy was exclusively to validate the PIROP ultrasound-based system for measuring peri-implant soft tissue thickness by evaluating measurement agreement with a direct clinical reference method. Accordingly, the analysis focused solely on methodological endpoints related to measurement validity and reliability.

All measurements included in this observational substudy were performed at predefined time points according to the parent study protocol. Only participants with complete paired measurements obtained using both the ultrasound-based system and the reference clinical method were included in the analysis. Procedure allocation from the parent randomized clinical trial was not considered at any stage of the present analysis, and no comparisons between randomized groups were performed.

As this manuscript reports a methodological validation substudy and does not present randomized treatment comparisons, participant flow between intervention arms, or clinical outcome data, CONSORT flow reporting was not applicable to the present analysis.

### 2.2. Ethical Approval

The study was conducted in accordance with the Declaration of Helsinki and approved by the Bioethics Committee of Wrocław Medical University, Poland (protocol code: KB-863/2021; date of approval: 28 October 2021). The study protocol, patient information sheet, and informed consent forms were reviewed and approved under this ethical approval, and all participants provided written informed consent prior to enrollment.

The present methodological validation substudy was conducted under the same ethical approval and informed consent framework as the parent investigator-initiated randomized clinical trial. The parent randomized clinical trial was registered at ClinicalTrials.gov (NCT07324187) and conducted at the Wrocław Medical University Dental Center (Medical Innovation Center Wrocław).

### 2.3. Study Population

Participants in the parent randomized clinical trial were eligible for inclusion in this methodological substudy. Inclusion and exclusion criteria were identical to those defined in the main study protocol, and no additional criteria were applied for the purposes of the present analysis.

Only participants with complete paired measurements obtained using both the PIROP ultrasound-based system and the reference clinical method were included.

The final sample consisted of 36 participants with 40 implant sites, 15 females and 21 males, with a mean age of 38.2 ± 9.1 years (range: 26–54 years). Each participant contributed one planned peri-implant measurement site.

Each measurement site was assessed at two predefined time points: at baseline and three months after healing. Each site–time point combination was treated as an independent measurement unit, resulting in a total of 80 paired measurements included in the analysis. For agreement analyses, measurements obtained at baseline (T0) and at the three-month follow-up (T1) were analyzed separately in order to preserve independence of observations within each time point.

### 2.4. Description of the PIROP Ultrasound-Based System

**Figure 1:**
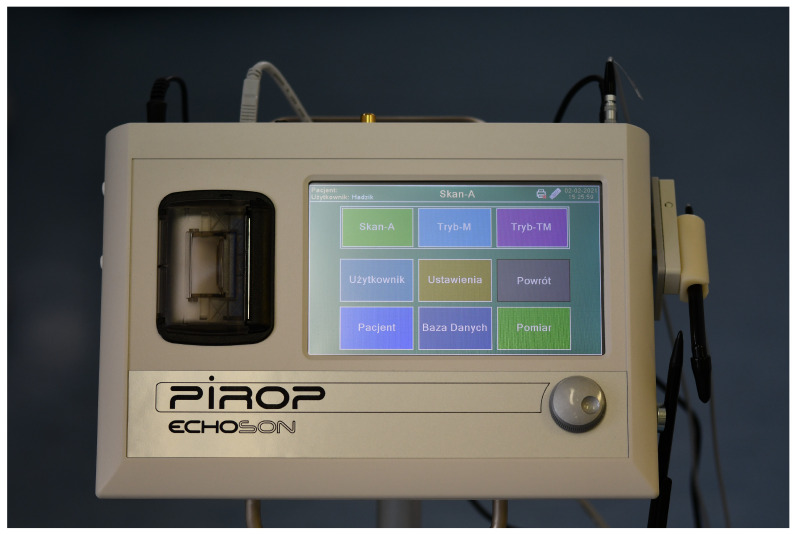
The PIROP-G ultrasound-based system (ECHOSON S.A., Puławy, Poland) used for non-invasive measurement of peri-implant soft tissue thickness in the present study.

The PIROP-G ultrasound biometric scanner (ECHOSON S.A., Puławy, Poland) is a high-frequency A-scan ultrasonic device designed for non-invasive measurement of soft tissue thickness in the oral cavity, including peri-implant soft tissues. The system is equipped with a dedicated intraoral probe operating at approximately 20 MHz and is capable of measuring soft tissue thickness in the range of 0.25 to 6 mm with an axial resolution of 0.01 mm (10 μm) (Figure 1).

The device interface uses a touch-screen display for user interaction, and the system provides automatic averaging of repeated measurements performed at a single site. In the present study, for each peri-implant site the PIROP-G performed a series of ten (10) consecutive measurements, and the mean value of these measurements was used for analysis.

Ultrasonic measurements in this study were performed by a single calibrated examiner according to a standardized measurement protocol.

The use of automated measurement acquisition and averaging is intended to improve precision and reduce random measurement variability inherent in single ultrasonographic readings.

### 2.5. Measurement Protocol and Reference Method

Peri-implant soft tissue thickness measurements were performed at a standardized measurement point located at the crest of the alveolar ridge at the planned implant site.

At baseline, measurements were obtained prior to implant placement and surgical incision. Ultrasonic measurement using the PIROP system was performed first, followed by a direct clinical measurement obtained after incision using a calibrated periodontal probe with 1 mm markings (UNC-15, Hu-Friedy, Chicago, IL, USA), which served as the reference clinical method.

The same measurement protocol was repeated three months later, following a period of closed healing and immediately before implant uncovering.

### 2.6. Examiner Calibration

Prior to the study, the examiner underwent calibration procedures to ensure consistency in both ultrasound-based and direct clinical measurements. Intra-examiner reliability was assessed by repeated measurements performed in a subset of cases.

### 2.7. Statistical Analysis

Statistical analysis was performed to evaluate agreement between measurements obtained using the PIROP ultrasound-based system and the reference clinical method. Relative differences between measurements obtained with the reference clinical method and the PIROP system were calculated as a descriptive measure using the following formula:

$$\mathrm{Relative difference}\;(\%)=\frac{A-B}{B}\times 100$$

where A represents the measurement obtained using the reference clinical method (periodontal probe), and B represents the corresponding measurement obtained using the PIROP ultrasound-based system. Negative values indicate that the reference clinical measurement yielded lower values compared with the PIROP measurement.

Agreement analyses were performed separately for baseline (T0) and three-month follow-up (T1) measurements to appropriately account for the repeated-measures structure of the data. Agreement between measurement methods was assessed using the intraclass correlation coefficient (ICC) based on a two-way mixed-effects model with absolute agreement, with ICC(3,1) calculated for single measurements and ICC(3,k) calculated for averaged measurements. A pooled analysis combining T0 and T1 measurements was additionally performed for descriptive and exploratory purposes only and was not used for inferential conclusions.

Agreement between methods was further evaluated using Bland–Altman analysis, including calculation of the mean difference (bias) and 95% limits of agreement (LoA). Separate Bland–Altman plots were generated for baseline (T0), three-month follow-up (T1), and pooled measurements to visually assess agreement across the measurement range.

Treatment allocation from the parent randomized clinical trial was not considered in the statistical analysis. All measurements were performed by the same calibrated examiner to ensure consistency of the measurement protocol. Statistical analyses were performed using R software (R Foundation for Statistical Computing, Vienna, Austria), with ICC calculated using the psych package.

## 3. Results

### 3.1. Study Sample and Measurements

A total of 36 patients were included in the present methodological validation analysis, contributing a total of 40 planned peri-implant measurement sites. Peri-implant soft tissue thickness measurements were performed at two predefined time points: the first measurement was obtained at baseline, prior to implant placement and surgical incision, and the second measurement was obtained three months later, following a period of closed healing and immediately before implant uncovering. Each site–time point combination was treated as an independent measurement unit.

In total, 80 paired measurements were obtained, each consisting of a measurement performed using the PIROP ultrasound-based system and a corresponding measurement obtained using the reference clinical method.

**Figure 2:**
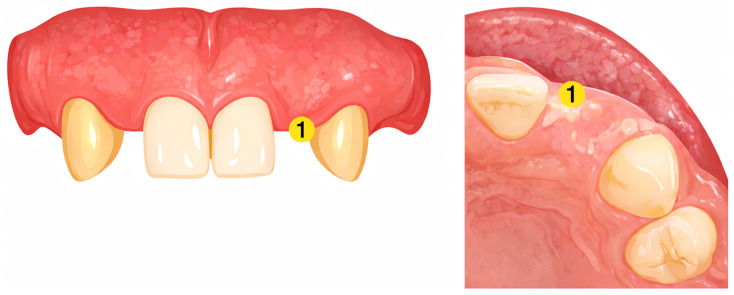
Schematic representation of the measurement site used for peri-implant soft tissue thickness assessment. Measurements were performed at the crest of the alveolar ridge at the planned implant site (marked as point 1).

**Figure 3:**
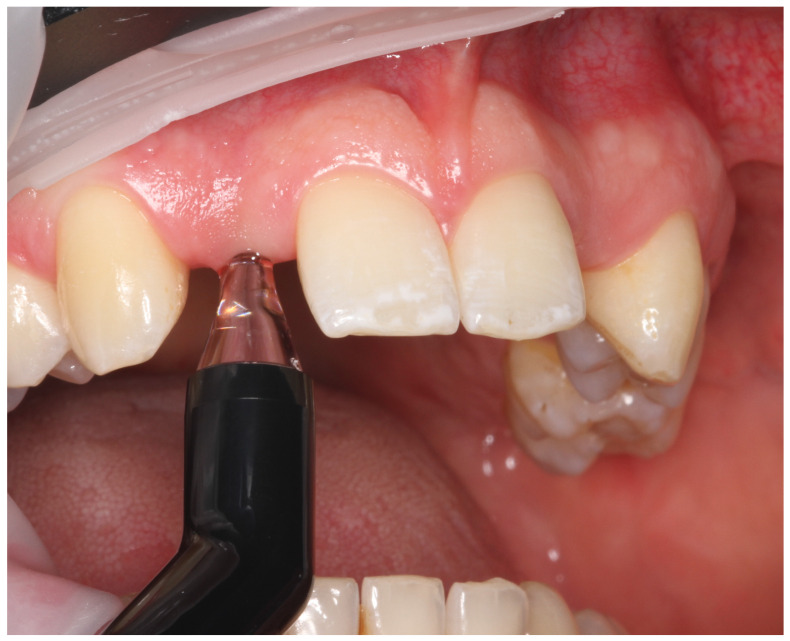
Intraoral photograph demonstrating ultrasound-based measurement of soft tissue thickness at an edentulous site in the maxillary anterior region.

All measurements were performed at a standardized measurement point located at the crest of the alveolar ridge at the implant site (Figure 2 and Figure 3).

### 3.2. Intraclass Correlation Coefficient (ICC) Analysis

Intraclass correlation analysis demonstrated a very high level of agreement between peri-implant soft tissue thickness measurements obtained using the PIROP ultrasound-based system and the reference clinical method at both evaluated time points.

At baseline (T0), the ICC for single measurements (ICC(3,1)) was 0.876 (F = 15.1, df_1_ = 39, df_2_ = 39, *p* = 3.43 × 10^−14^), indicating excellent reliability for individual paired measurements. The ICC for averaged measurements (ICC(3,k)) was even higher, reaching 0.934 (F = 15.1, df_1_ = 39, df_2_ = 39, *p* = 3.43 × 10^−14^), reflecting excellent agreement when multiple measurements were averaged.

At the three-month follow-up after closed healing (T1), similarly high agreement was observed. The ICC for single measurements (ICC(3,1)) was 0.864 (F = 13.7, df_1_ = 39, df_2_ = 39, *p* = 1.77 × 10^−13^), while the ICC for averaged measurements (ICC(3,k)) was 0.927 (F = 13.7, df_1_ = 39, df_2_ = 39, *p* = 1.77 × 10^−13^).

Overall, ICC analysis confirmed excellent agreement between the ultrasound-based and reference clinical measurements at both time points, with particularly strong reliability observed for averaged ultrasonic measurements.

### 3.3. Agreement Between PIROP and Reference Method

Relative differences between peri-implant soft tissue thickness measurements obtained using the PIROP ultrasound-based system and the reference clinical method are presented in Table 1.

At baseline (T0), the mean relative difference between the two methods was −3.41% (SD: 15.75%), with a median difference of −5.93% (interquartile range: −13.04% to 0.00%). The Wilcoxon signed-rank test revealed a statistically significant difference between the paired measurements obtained with the two methods (*p* = 0.0057).

**Figure 4:**
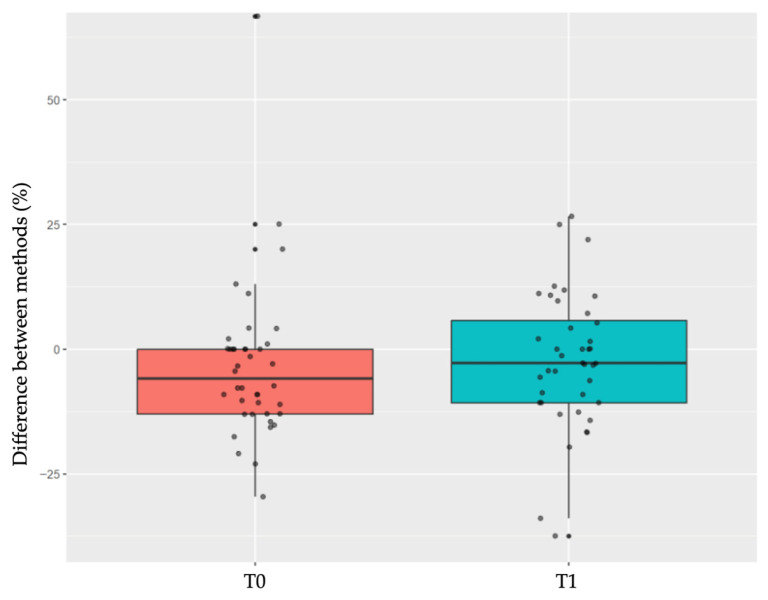
Distribution of relative differences between peri-implant soft tissue thickness measurements obtained using the PIROP ultrasound-based system and the reference clinical method at baseline (T0) and at the three-month follow-up after closed healing (T1). Boxplots represent the median and interquartile range, with individual paired measurements shown as points.

At the three-month follow-up after closed healing (T1), the mean relative difference was −2.21% (SD: 13.54%), with a median difference of −2.85% (interquartile range: −10.71% to 6.20%). No statistically significant difference between the paired measurements was observed at this time point (*p* = 0.214), Figure 4.

### 3.4. Bland–Altman Analysis

Bland–Altman plots were used to visually and quantitatively assess agreement between the PIROP ultrasound-based system and the reference clinical method across the range of measured peri-implant soft tissue thickness values.

**Figure 5:**
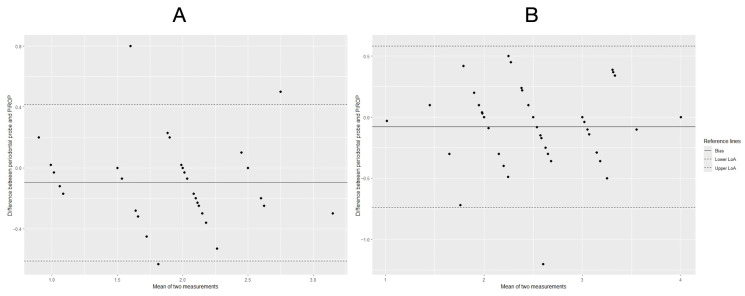
Bland–Altman plots illustrating agreement between peri-implant soft tissue thickness measurements obtained using the PIROP ultrasound-based system and the clinical reference method with a periodontal probe at baseline (T0) (**A**) and after three months of healing (T1) (**B**). The horizontal axis represents the mean of the two measurements, while the vertical axis shows the difference between methods (periodontal probe—PIROP). The solid line indicates the mean difference (bias), and the dashed lines represent the 95% limits of agreement (LoA). For both time points, most paired observations fall within the limits of agreement, indicating good agreement between methods without relevant systematic measurement error across the measurement range. At baseline (T0), the mean bias was −0.10 mm, with 95% limits of agreement ranging from −0.61 mm to 0.42 mm; at the three-month follow-up (T1), the mean bias was −0.08 mm, with limits of agreement from −0.74 mm to 0.58 mm.

At both baseline (T0) and the three-month follow-up (T1), the mean differences (bias) between the two measurement methods were small and close to zero, indicating the absence of relevant systematic measurement error. The majority of individual paired measurements fell within the 95% limits of agreement, demonstrating acceptable dispersion and consistent agreement across the measurement range (Figure 5).

Separate Bland–Altman analyses performed for T0 and T1, as well as for pooled data, revealed no evidence of proportional bias, as differences between methods did not increase with increasing mean tissue thickness values. Although individual outliers were observed, their distribution was random and did not suggest time-point–dependent or thickness-dependent systematic error.

Taken together, Bland–Altman analysis confirmed good agreement between the PIROP ultrasound-based system and the direct clinical reference method, supporting the validity of ultrasound-based assessment for quantitative measurement of peri-implant soft tissue thickness under standardized clinical conditions.

## 4. Discussion

The present study was designed as a prospective methodological validation study to assess the agreement between an ultrasound-based measurement system and a direct clinical reference method for the assessment of peri-implant soft tissue thickness. The analysis focused exclusively on methodological endpoints and did not aim to evaluate procedure-related outcomes. This approach allowed for a controlled comparison of two measurement techniques under standardized clinical conditions. The clinical relevance of accurately assessing peri-implant soft tissue thickness is underscored by the prospective controlled trial by Linkevicius et al. [6], which demonstrated significantly greater marginal bone loss around implants placed in sites with mucosal thickness ≤ 2 mm compared with thicker tissues.

The results demonstrated small relative differences between measurements obtained using the PIROP ultrasound-based system and those derived from the reference clinical method based on surgical incision and periodontal probing. Differences between the two methods were more pronounced at baseline, whereas no statistically significant differences were observed at the follow-up examination after healing. In line with the predefined aim and null hypothesis, the observed levels of agreement between ultrasound-based and direct clinical measurements did not reveal relevant systematic discrepancies between methods.

Although changes in soft tissue thickness between baseline and follow-up may be of clinical interest, the present study was not designed to evaluate treatment-related tissue alterations, and therefore no conclusions regarding surgical effects on tissue thickness can be drawn from these data. These findings suggest that ultrasound-based assessment provides measurements that are generally comparable to those obtained using a direct clinical approach. The excellent intraclass correlation coefficients observed at both time points, together with narrow and clinically acceptable limits of agreement in Bland–Altman analysis, indicate that the PIROP ultrasound-based system provides measurements that are highly consistent with direct clinical assessment and suitable for use in longitudinal clinical studies requiring repeated soft tissue measurements.

Assessment of peri-implant soft tissue thickness has traditionally relied on invasive clinical techniques. These include transgingival probing methods performed without surgical incision, such as soft tissue perforation using periodontal probes or endodontic instruments (e.g., files) advanced until bone contact is achieved. Transgingival probing performed without surgical incision was popularized by early clinical studies evaluating peri-implant mucosal dimensions, most notably the work of Kan et al., who applied direct soft tissue perforation to quantify peri-implant mucosal thickness in humans [12]. Although such approaches allow estimation of tissue thickness in millimeters, they are influenced by several operator- and tissue-related factors, including probe angulation, insertion force, tissue compression, and the use of local anesthesia. Other invasive methods involve direct measurement following surgical incision, which enables visual assessment of tissue dimensions and can regarded as a reference approach. When the incision is performed at the crest of the alveolar ridge, the flap is fully elevated, and tissue thickness is measured directly using a millimeter probe, the potential for systematic measurement error is minimal. Under these conditions, the primary sources of bias are limited to the operator’s visual judgment and the inherent difficulty in discerning small differences in tissue thickness, particularly in the case of thin soft tissue flaps. In the prospective clinical trial by Linkevicius et al., soft tissue thickness was assessed intraoperatively following surgical incision using a periodontal probe, allowing direct quantitative measurement of the mucosal thickness prior to implant placement [20].

In certain clinical situations, soft tissue thickness has also been indirectly estimated based on the height or exposure of healing abutments; however, this approach remains indirect and dependent on prosthetic and surgical variables rather than direct tissue measurement.

Radiographic techniques have also been used for the assessment of peri-implant soft tissue thickness. Cone beam computed tomography (CBCT) allows cross-sectional visualization of edentulous sites and simultaneous evaluation of hard and soft tissue structures [13,21]. Nevertheless, accurate soft tissue measurement using CBCT requires careful separation of soft tissues from adjacent anatomical structures, such as the lips or cheeks, which is not always achievable under routine imaging conditions. Furthermore, the limited soft tissue contrast and spatial resolution of CBCT, particularly in sites with thin mucosa, may reduce measurement accuracy. Image artifacts and partial volume effects may further compromise precision. Importantly, CBCT-based assessment involves additional radiographic exposure, which limits its applicability for repeated measurements or longitudinal studies focused primarily on soft tissue evaluation.

Non-invasive qualitative methods have been proposed as alternatives to invasive and radiographic techniques. Color-coded probes, such as the Colorvue probe, are based on the assessment of tissue translucency or probe visibility through the mucosa [14,15,22]. These approaches allow chairside, non-invasive evaluation and may assist in soft tissue phenotype classification. However, they provide only qualitative or categorical information and do not allow for direct quantitative measurement of soft tissue thickness expressed in millimeters. As a result, their use is limited when precise and reproducible quantitative assessment is required.

Ultrasound-based techniques represent a non-invasive approach capable of providing direct quantitative measurements of soft tissue thickness. High-frequency ultrasonography enables real-time evaluation of soft tissue dimensions without tissue penetration or radiographic exposure, making it particularly suitable for repeated measurements in both clinical research and longitudinal study designs.

An early prototype of the PIROP ultrasonic device was previously described by Bednarz, who presented its technical concept and potential application for periodontal soft tissue thickness assessment [16]. However, this initial report was exploratory in nature and focused primarily on technical feasibility, without providing formal validation against a reference measurement method. Subsequently, Gánti et al. [17] evaluated the reproducibility of the PIROP ultrasonic biometer for gingival thickness measurements in a clinical setting. In that study, repeated ultrasound measurements were compared with an invasive transgingival probing technique using an endodontic spreader as a reference. The authors reported acceptable intra-examiner reproducibility and repeatability of ultrasonic measurements, supporting the technical reliability of the method. Nevertheless, the scope of that investigation was limited to reproducibility assessment and did not include a comprehensive agreement analysis or validation under peri-implant clinical conditions. Ultrasound-based techniques have also been applied in randomized clinical trials evaluating peri-implant soft tissue augmentation. Puzio et al. employed ultrasound measurements to assess soft tissue thickness changes around implants placed in the aesthetic zone following connective tissue grafting or the use of a xenogeneic collagen matrix during a one-year randomized follow-up [23]. In this study, ultrasonography served as a non-invasive outcome assessment tool for monitoring soft tissue changes over time. However, ultrasound measurements were not formally validated against a direct clinical reference method, and the primary focus remained on treatment outcomes rather than methodological validation. Similarly, in Hadzik et al. and Ciszyński et al., ultrasound measurements were used to evaluate peri-implant soft tissue stability following soft tissue augmentation procedures [18,24]. Although these findings support the feasibility and clinical usefulness of ultrasound-based assessment, they further highlight that the method has predominantly been used as an outcome measurement tool rather than being rigorously validated as a diagnostic modality for peri-implant soft tissue thickness assessment.

The present study builds upon this methodological background by extending ultrasound-based assessment to a peri-implant clinical setting and by directly validating the PIROP ultrasound-based system against a clinical reference method based on surgical incision and calibrated periodontal probing. By focusing on measurement agreement at predefined clinical time points, this study addresses a critical methodological gap and provides a validated framework for the use of ultrasound-based measurements in future randomized clinical trials and longitudinal studies evaluating peri-implant soft tissue changes.

A comparative overview of currently available methods for peri-implant soft tissue thickness assessment is provided in Table 2, highlighting the methodological advantages and limitations of each approach.

Beyond peri-implant applications, the validated ultrasound-based measurement approach may have broader relevance for clinical research involving oral soft tissues. The non-invasive and quantitative nature of ultrasonography makes it particularly suitable for studies assessing gingival recession, periodontal phenotype characterization and modification, as well as soft tissue thickening procedures using autogenous grafts or various biomaterials. In the context of orthodontic treatment planning, non-invasive assessment of gingival and mucosal thickness may support risk evaluation for treatment-induced recession and facilitate biologically driven planning of tooth movement, particularly in patients with a thin soft tissue phenotype. From a biomaterials research perspective, ultrasound-based measurements may facilitate standardized evaluation of soft tissue responses to different grafting materials and matrices, enabling objective comparison across clinical studies. In addition, ultrasonography may be applied in observational and epidemiological studies investigating gingival thickness distribution and its association with demographic or anatomical variables, as well as in longitudinal pre–post treatment designs requiring repeated, standardized assessment of soft tissue dimensions. Importantly, the ability to perform repeated measurements without surgical intervention or radiographic exposure represents a methodological advantage in studies focusing on dynamic soft tissue changes over time and contributes to improved methodological harmonization across clinical trials.

This study has several limitations that should be acknowledged. First, it was designed as a methodological validation substudy nested within a randomized clinical trial and was intentionally restricted to the assessment of measurement agreement between methods. Consequently, procedure-related outcomes and biological changes in soft tissue thickness were not evaluated.

Second, all measurements were performed by a single calibrated examiner. While this approach ensured strict standardization of the measurement protocol and minimized operator-related variability, it did not allow assessment of inter-examiner reliability, which should be addressed in future studies.

Third, measurements were obtained at predefined time points according to the parent study protocol, which limited evaluation of additional healing stages. Although measurements were collected at two time points per patient, agreement analyses were intentionally performed separately for each time point to preserve independence of observations. The pooled Bland–Altman analysis should therefore be interpreted with caution, as it does not explicitly model within-subject correlation and was included for descriptive purposes only.

Finally, the reference method was based on direct measurement following surgical incision using a calibrated periodontal probe, which is widely regarded as the most accurate clinical approach for assessing soft tissue thickness. Nevertheless, as with any manual measurement technique, minimal variability related to visual reading of the millimeter scale cannot be entirely excluded, particularly in sites with thin soft tissues.

Despite these limitations, the standardized study design and paired-measurement approach support the methodological validity of the present findings.

## 5. Conclusions

Within the limitations of this prospective methodological validation study, the PIROP ultrasound-based system demonstrated good agreement with direct clinical measurements obtained following surgical incision and calibrated periodontal probing for the assessment of peri-implant soft tissue thickness. The observed differences between the two methods were small and clinically acceptable, supporting the reliability of ultrasound-based assessment under standardized clinical conditions.

## Figures and Tables

**Table 1:** Relative differences between peri-implant soft tissue thickness measurements obtained using the PIROP ultrasound-based system and the reference clinical method at baseline (T0) and three-month follow-up (T1).

Time Point	N	Mean Relative Difference (%) ± SD	Median Relative Difference (%)	Interquartile Range (%)	*p*-Value †
T0 (baseline)	40	−3.41 ± 15.75	−5.93	−13.04 to 0.00	0.0057
T1 (3 months)	40	−2.21 ± 13.54	−2.85	−10.71 to 6.20	0.214

† Wilcoxon signed-rank test comparing paired measurements obtained using the PIROP ultrasound-based system and the reference clinical method.

**Table 2:** Comparison of methods for peri-implant soft tissue thickness assessment.

Method	Invasiveness	Quantitative (mm)	Repeatability	Typical Applications	Main Limitations
Transgingival probing (no incision)	Yes	Yes	Limited	Periodontal phenotype assessment	Tissue compression, operator-dependent
Measurement after surgical incision	Yes	Yes	High	Reference clinical measurements	Invasive, single time point
CBCT-based assessment	No	Yes	Moderate	Pre-implant site analysis	Radiation exposure, low soft tissue contrast
Color-coded probes (Colorvue)	No	No (qualitative)	Moderate	General Phenotype classification	Categorical assessment only
Ultrasound-based measurement (PIROP)	No	Yes	High	Peri-implant studies, periodontology, biomaterials, epidemiology, orthodontic treatment planning	Requires operator training

## Data Availability

The data presented in this study are available from the corresponding author upon reasonable request. The data are not publicly available due to ethical and privacy restrictions related to the protection of personal data of the study participants.
